# Generalized joint hypermobility, scoliosis, patellofemoral pain, and physical abilities in young dancers

**DOI:** 10.1186/s12891-021-04023-z

**Published:** 2021-02-09

**Authors:** Nili Steinberg, Shay Tenenbaum, Aviva Zeev, Michal Pantanowitz, Gordon Waddington, Gali Dar, Itzhak Siev-Ner

**Affiliations:** 1 Anatomy Laboratory, The Academic College at Wingate, Wingate Institute, Netanya, Israel; 2 Department of Orthopedic Surgery, Chaim Sheba Medical Center Tel-Hashomer, affiliated with the Sackler Faculty of Medicine, Tel Aviv University, Tel Aviv, Israel; 3 Faculty of Health, University of Canberra, Canberra, Australia; 4 Department of Physical Therapy, Faculty of Social Welfare & Health Studies, University of Haifa, Haifa, Israel; 5 Orthopedic Rehabilitation Department, Chaim Sheba Medical Center, Tel-Hashomer, Israel

**Keywords:** Generalized joint hypermobility, Scoliosis, PFP, Strength ability, Postural balance

## Abstract

**Background:**

Many young girls with generalized joint hypermobility (GJH) choose to participate in dance because their bodies are suited for this activity. Scoliosis tends to occur often in thin girls, who also are more likely to choose dance. Both anomalies (GJH and scoliosis) may be related to reduced abilities such as diminished strength and insufficient postural balance, with increased risk for musculoskeletal conditions. The main objectives of the present study were to determine the prevalence of dancers with GJH, the prevalence of dancers with scoliosis, and the prevalence of dancers with these two anomalies; and, to determine differences in physical abilities and the presence of patellofemoral pain (PFP) between young female dancers with and without such anomalies.

**Methods:**

One hundred thirty-two female dancers, aged 12–14 years, were assessed for anthropometric parameters, GJH, scoliosis, knee muscle strength, postural balance, proprioception ability, and PFP.

**Results:**

GJH was identified in 54 dancers (40.9%) and scoliosis in 38 dancers (28.8%). Significant differences were found in the proportion of dancers with no anomalies (74 dancers, 56.1%) and dancers with both anomalies (34 dancers, 25.8%) (*p* < .001). Dancers with both anomalies had reduced dynamic postural balance in the anterior direction (*p* = .023), reduced proprioception ability (*p* < .001), and weaker knee extensors (*p* = .036) and flexors (*p* = .040) compared with dancers with no anomalies. Among dancers with both anomalies, 73.5% suffered bilateral PFP, 17.6% suffered unilateral PFP, and 8.8% had no PFP (*p* < .001).

**Conclusions:**

A high prevalence of young girls participating in dance classes had GJH, as the increased joint flexibility probably provides them with some esthetic advantages. The high prevalence of scoliosis found in these young dancers might be attributed to their relatively low body mass, their delayed maturation, and the selection process of dancers. Dancers with both GJH and scoliosis had decreased muscle strength, reduced postural balance, reduced proprioception, with higher risk of PFP. The main clinical implications are the need to reduce the risk of PFP among dancers by developing appropriate strength and stabilizing exercises combined with proprioceptive and postural balance training, to improve the correct alignment of the hyperextended and hypermobile joints, and to improve their supporting muscle strength.

## Background

Generalized joint hypermobility (GJH), a requirement for many dance styles, is a common physiological trait of many young dancers [1]. GJH is characterized by increased joint flexibility, where the joints move beyond the “normal” limits [2]. This characteristic has mostly been assessed by the Beighton score [3]. GJH was reported to be dependent on the age, sex, and maturity of the individuals [4]. It was found to be more common in young subjects and in females, and this prevalence varies with ethnic background [2, 5]. The high prevalence of GJH among dancers might be due to some self-selection of body type, with dancers who could not match what is perceived to be the “perfect” dance movements performed by their hypermobile counterparts dropping out [6, 7]. It is generally agreed that GJH is associated with many manifestations that lead to reduced abilities, such as altered proprioception, increased perception of pain, lower muscle strength, autonomic dysfunction, chronic pain disorders, joint dislocation, sporting injuries, and musculoskeletal disorders [8, 9]. A systematic review of sport participants supported a link between GJH and lower limb injury incidence and prevalence [10], mainly to the knee joint [11]. As the knee relies almost completely on the passive ligamentous and capsular restraints, athletes with GJH may rely mostly on their dynamic muscular control to maintain joint stability of their hyperextended joint. That in turn may put the GJH athletes at a greater risk for soft tissue injury.

Adolescent idiopathic scoliosis is a 3-dimensional, structural curvature of the spine, with lateral and rotational components, occurring at pubescence without a definitive cause [12]. Initial evaluation in dancers should involve a focused history and physical examination, with the Adam’s forward bend test and Magee’s “skyline” view test being particularly useful for detection of scoliosis [13] in the dance studio [14]. Scoliosis is reportedly a common clinical presentation in young dancers [14], with a high incidence in dancers compared to their age-matched controls [15]. Scoliotic dancers were described as having a high tendency to be taller, with delayed menarche, low body mass index, and higher prevalence of secondary amenorrhea, compared with non-scoliotic dancers of the same age cohorts [16]. Furthermore, scoliosis is more common in the ectomorphic body composition, in GJH dancers and in dancers with abnormal dietary characteristics [17]. The high prevalence of scoliotic dancers with these common and desired characteristics are probably the end result of a rigorous selection process [6, 18]. Other physical features such as reduced muscle strength [19] and reduced postural balance [20] were found to be associated with scoliosis, yet these studies reported only on adolescent non-dancer populations.

During childhood and adolescence, which are the critical periods of growth and maturation, many girls practice dancing. Of main concern is the question of whether girl with one of these two different anatomical anomalies (GJH and scoliosis) have greater chance to have the second anomaly as well. A previous study suggested that GJH might be considered a contributing factor in scoliosis as a result of altered collagen structure and cross-linking [18]. As these two anomalies are common and traditionally desired in dancers, a high prevalence of dancers with both those anomalies are expected.

Another question is whether dancers with scoliosis and with GJH are at higher risk to develop patella-femoral pain (PFP). Among dancers, weak tendons and weak joint structure – conditions that refer to GJH, may slow the response of the soft tissues to training effects, making them more vulnerable to musculoskeletal conditions during training or performance, as well as to prolonged periods of post-injury recovery [1, 9]. Considering scoliosis and injuries, female dancers with scoliosis were reported to demonstrate a higher prevalence of musculoskeletal injuries compared to the non-scoliotic girls [14]. The prolonged growth spurts among the scoliotic dancers were suggested to be related to this increased risk for injuries [14, 16, 21]. It should be acknowledged that the stresses exerted on the hyperextended joints and the spine curvature over the long hours of practice along years of training, may also be associated with an increased incidence of musculoskeletal condition among young dancers [1, 9, 22, 23]. The rationale for measuring only patellofemoral pain (PFP) among our dancers in the current study (regardless of other conditions) was that GJH and scoliosis were reported to be related to PFP [24]. Furthermore, PFP was found to be the most prevalent musculoskeletal condition among young adolescent dancers along the growth-spurt period [25]. In addition, young dancers suffering from PFP were found to have a significantly greater prevalence of scoliosis, a higher prevalence of hypermobility of the patella in knee extension and a higher prevalence of hypermobility of the patella in 30° knee flexion – compared with dancers with no-PFP [26].

The main objectives of the present study were to determine the prevalence of dancers with GJH, the prevalence of dancers with scoliosis, and the prevalence of dancers with these two anomalies; and, to determine differences in physical abilities and the presence of PFP between those with and those without such anomalies in young female dancers.

## Methods

In this cross-sectional study, a group of 132 female dancers aged 12–14 years were recruited. The sample was selected based on convenience, with the dancers attending three different schools with the same special dance program [27]. The research was approved by the Hospital Human Subjects Review Board in accordance with the Helsinki Declaration. Approval was also obtained from the Ministry of Education and each school’s administration. A consent form was signed by each participant and one of her parents, and the rights of the dancers were protected. The government-funded dance schools offered high quality dance training. The curriculum of the program was based on a minimum of 10 h/week of practical dance lessons (classical dance, modern dance, and composition), and a minimum of 5 h/week of academic lessons (dance history, anatomy, kinesiology, and music) [28]. To be included in the study, a dancer was required to be a full-time student in the dance program; to be fully active in all dance classes over the 3 months’ prior the study and along study; and, not to have been absent from dance classes due to pain/discomfort/ injury for more than 3 days over the last 3 months. Dancers with a history of knee surgery, dancers who were not fully active in dance classes during the 3 months prior to the study and during the study, or dancers who took an absence of more than 3 days from dance classes due to pain/discomfort/injury in the last 3 months, were excluded from the study [28].

The dancers were interviewed for age, age at onset of menarche, and age at onset of dance practice; and, for total number of hours per week (h/week) of dance practice and total h/week of *en-pointe* dancing. Dancers were requested to report their pubertal development in a self-report questionnaire (Tanner stage questionnaire) [29].

### Clinical measurements

All measurements for all participants were taken three times, with the average result used for analysis. For more details about the measurements, please see [14, 27, 30].

#### Blinding

All assessors were blinded to the results of any of the other measures.

### Body physique measures

Standing height (m) and weight (kg) measurements were taken using standard anthropometric tools. BMI was calculated (kg/m^2^). Leg length (cm) was measured from the anterior superior iliac spine to the distal end of the medial malleolus.

### Generalized joint hypermobility (GJH)

GJH was assessed by the Beighton 9-Point Hypermobility Test, following previous publications [25, 31]. A dancer was diagnosed with GJH if she had a Beighton score of ≥5/9 [25, 31].

### Scoliosis

To identify scoliosis, the Adams forward-bend test [32] and Magee’s “skyline” view test [33] were used by one of the authors (S.I. – an orthopedic surgeon, specializing in dance medicine; had screened over 3000 dancers in the last several decades) for all the dancers. First, all the dancers underwent the Adams forward-bend test. When the Adams forward-bend test indicated a positive clinical diagnosis, the dancer was referred to a body posture test (Magee’s “skyline” view test), similar to a previous publication on dancers [14]. Following these tests, the inclusion criterion for the scoliosis group was determined: a positive Adams forward-bend test, with deviation/s from the normal posture (Magee’s “skyline” view test) that support/s a structural scoliosis.

### PFP assessment

Each dancer was examined for PFP, similar to previous publications on dancers [25, 27, 34]. The assessment was performed by one of two physicians (both were orthopedic surgeons specializing in dance medicine) [25]. The criteria for PFP diagnosis were as follows: knee pain complaints (around and/or in the retropatella region), Visual Analogue Scale (VAS) ≥3 during dance activities; pain aggravated during different activities loading the knees (e.g. descending stairs, jumping) and affecting their dancing.

### Dynamic postural balance (DPB)

The Y balance test for each leg was performed (using a commercially available device: Y Balance Test, Move2Perform, Evansville, IN), by a professional sport physical therapist, similar to previous publications [27, 35]. After the dancers had completed four practice trials in each direction on each leg, they were given a 2-min rest period, and then performed three test trials in each direction (distance measured in cm). A composite score was calculated using the following formula: the sum of the maximum reach achieved in each of the three directions/leg length X3. A lower score represents impaired DPB.

### Proprioception ability

**Fig. 1:**
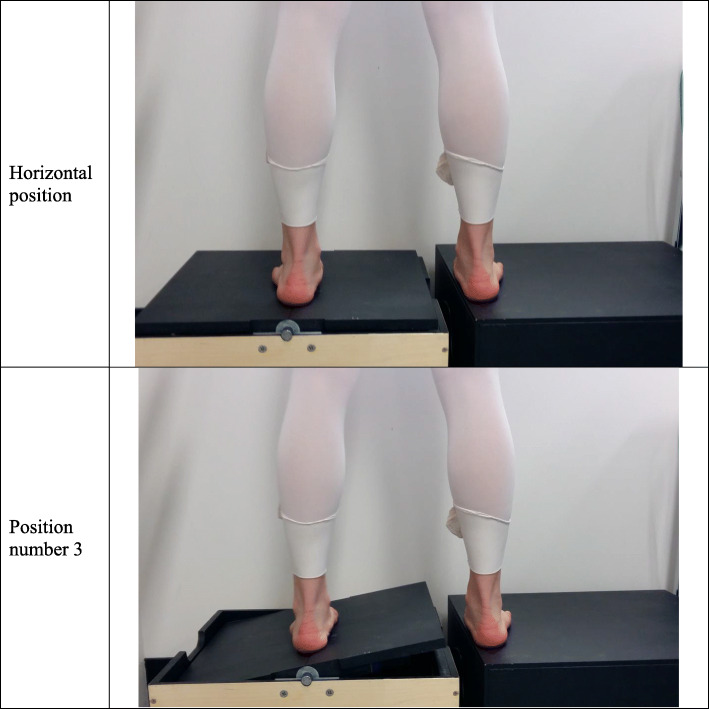
The AMEDA device using for discrimination of the extent of ankle inversion movement has five inversion movements, in the order from the smallest (position 1) through to the largest (position 5). The five ankle positions are 8, 9, 10, 11, and 12 degrees of inversion, and controlled by the computer-controlled stepper motor device. The participants underwent 50 test trials, presented without feedback and in a random sequence

Ankle inversion movement sensitivity was tested on the non-dominant leg using the Active Movement Extent Discrimination Apparatus (AMEDA), similar to previous publications on dancers [27, 36] (Fig. 1). This device examines the individual’s ability to discriminate between differences in the amount of ankle inversion movements during a normal weight-bearing stance. The protocol for testing consisted of a standardized familiarization session followed by a data collection session based on a previously published study [37]. The ability to discriminate between adjacent angles was calculated by generating a receiver operating characteristic (ROC) curve for each paired comparison, and calculating an area under the curve (AUC) for each of the four paired comparisons. The participant’s proprioceptive ability score is the mean AUC that give scores between 0 and 1, where a score of 1 is equivalent to simple chance. A higher score represents better proprioception ability. For more details, please see [37].

### Strength ability

Muscle force measured in Newton (N) using a hand-held Dynamometry (HHD) (MicroFET2TM, Hoggan Scientific LLC., Salt Lake City, UT) with a 4 cm-wide transducer pad. All muscle strength tests were performed by the same tester, a professional sport physical therapist. Strength ability tests for knee extensors and for knee flexors were performed, following a previous study on dancers [38]. The dancer sat on the edge of a bed with her feet not touching the floor, and her arms crossed in front of the body. The dancers were asked to keep their buttocks on the bed, and to extend the knee joint without a swing movement. The transducer was placed on the distal part of the leg (anteriorly for knee extensors and posteriorly for knee flexors). No warm-up was performed prior to testing. A higher score represents better muscle strength ability.

### Reliability tests

Intra- and inter-tester reliability tests were performed prior to data collection on 20 dancers. Intra-tester reliability tests were performed only for the examiner who performed the HHD strength measurements, the GJH assessment, and the YBT measurements. The examinations were performed twice by the same examiner, 3–5 days apart. An inter-tester reliability test was performed between the two examiners who assessed PFP (two physicians), conducting the measurements using the same method and blinded to each other’s results.

Both reliability indices (Kappa and ICC – intra-class correlation coefficient) produced good reliability results. Intra-tester reliability for scoliosis was determined for one author (S.I.); the Kappa value for the prevalence of scoliosis was 0.85. For intra-tester agreement ICC (95% CI): DPB A-P, ICC = .959 (.899–.983); DPB P-L, ICC = .936 (.846–.974); DPB P-M, ICC = .936 (.846–.974); and, for knee extension strength ICC = .950 (.880–.980); knee flexion strength ICC = .896 (.756 .957). The Kappa value for the presence of PFP was .806 (percent agreement = 90%) and the Kappa value for the prevalence of GJH was .864 (percent agreement = 92%). Inter-class correlation coefficient generated by the AMEDA (AUC score) tests was determined as ranging from 0.82–0.96.

### Data analysis

Dancers with GJH were compared with dancers with no GJH using a t-test and a chi-square test (for categorized parameters). The same tests were used for the dancers with scoliosis vs. dancers with no-scoliosis, and for dancers with both GJH and scoliosis vs. dancers with no-anomaly. CIs are represented for means and for PFP frequencies. Statistical analyses were performed using SPSS 25.0 (IBM Corp. Released 2017. IBM SPSS Statistics for Windows, Version 25.0. Armonk, NY: IBM Corp); *p* value was set at < 0.05.

## Results

Anthropometric parameters, age at menarche, pubic/breast Tanner score, dance background, and intensity of training and anatomical anomalies appear in Table 1. GJH was identified in 40.9% (54/132) of the dancers. Significant differences were found between the proportions of dancers with GJH and dancers with no GJH (*p* < .001). When comparing the GJH dancers with the non-GJH dancers, the GJH dancers were found to have significantly reduced dynamic postural balance in the anterior direction (55.6 ± 5.1 vs. 57.9 ± 5.3; *p* = .014) and significantly lower proprioception ability (.593 ± .035 vs. .640 ± .037; *p* < .001), compared with the non-GJH dancers. There were no statistically significant differences between those with and without GJH on any of the other outcome measures (Table 2).

**Table 1:** Anthropometric parameters, age at menarche, pubic/breast Tanner score, dance background and intensity of training and anatomical anomalies (*n* = 132)

		Mean/percentage	SD
**Anthropometric parameters and pubertal stage**	Height (m)	156.79	7.26
	Weight (Kg)	47.58	8.47
	BMI (Kg/m^2^)	19.23	2.43
	Leg length (cm) RT	83.80	4.37
	Leg length (cm) LT	84.03	4.35
	Age onset of menarche (Years)	12.55	.85
	Percentage of breast Tanner≥3–5	77.3%	
	Percentage of pubic Tanner≥3–5	73.5%	
**Dance background and intensity of training**	Number of hours per week last year	10.85	5.23
	Number of hours per week this year	13.65	5.04
	Average number of hours per day	2.83	1.25
	Onset classic ballet (years)	7.25	3.28
	Onset other style (years)	7.19	2.81
	Onset *En-pointe* (years)	12.08	1.74
	Number of hours per week *En-pointe*	1.98	1.20
**Anatomical anomalies**	GJH	40.9%	
	Scoliosis	28.8%	
	Both GJH and Scoliosis	25.8%

**Table 2:** Anthropometric parameters, postural balance and proprioception muscle strength measurements in dancers with GJH and with no-GJH

	Dancers with no- GJH (***n*** = 78)	Dancers with GJH (***n*** = 54)	***p*** value	Mean Difference	95% CI	
**Anthropometric parameters**					Lower	Upper
BMI (Kg/m^2^)	18.9 ± 2.5	19.6 ± 2.3	.153	−.62	−1.48	.23
**Postural balance and proprioception**						
YBT Anterior (cm)	57.9 ± 5.3	55.6 ± 5.1	**.014**	2.25	**.27**	**4.23**
YBT Post. Med. (cm)	86.9 ± 7.1	85.8 ± 7.9	.410	1.12	−1.56	3.81
YBT Post. Lat. (cm)	86.6 ± 10.7	85.5 ± 7.9	.510	1.05	−2.18	4.30
YBT composed score (cm)	93.2 ± 8.8	90.3 ± 8.2	.060	2.85	−.12	5.84
AMEDA (AUC score)	.640 ± .037	.593 ± .035	**<.001**	.048	**.031**	**.061**
**Muscle strength measurements**						
Knee extension (N)	241.6 ± 49.3	229.2 ± 52.4	.178	12.49	−5.74	30.72
Knee flexion (N)	121.0 ± 21.0	117.3 ± 19.8	.306	3.75	−3.48	10.98

*GJH* Generalized joint hypermobility, *BMI* Body mass index, *YBT anterior* Y balance test in the anterior direction, *YBT Post. Med.* Y balance test in the posterio-medial direction, *YBT Post. Lat.* Y balance test in the postero-lateral direction, *AMEDA* Active Movement Extent Discrimination Apparatus

Scoliosis was identified in 28.8% (38/132) of the dancers. The scoliotic dancers were found to have significantly weaker knee extensors and significantly weaker knee flexors compared with the non-scoliotic dancers (227.4 ± 52.7 vs. 251.2 ± 44.3; *p* = .017 and 116.2 ± 20.5 vs. 125.3 ± 18.5; *p* = .022, respectively). Furthermore, the scoliotic dancers were found to have significantly reduced proprioception ability compared with the non-scoliotic dancers (.603 ± .040 vs. .628 ± .042; *p* = .003). There were no statistically significant differences between those with and without scoliosis on any of the other outcome measures (Table 3).

**Table 3:** Anthropometric parameters, postural balance and proprioception muscle strength measurements in dancers with scoliosis and with no-scoliosis

	Dancers with no-scoliosis (***n*** = 94)	Dancers with scoliosis (***n*** = 38)	***p*** value	Mean Difference	95% CI	
**Anthropometric parameters**					Lower	Upper
BMI (Kg/m^2^)	19.0 ± 2.5	19.7 ± 2.2	.141	−.69	−1.63	.23
**Postural balance and proprioception**						
YBT Anterior (cm)	58.2 ± 6.5	56.2 ± 5.4	.075	2.01	−.20	4.24
YBT Post. Med. (cm)	87.3 ± 7.2	85.8 ± 7.7	.315	1.48	−1.43	4.40
YBT Post. Lat. (cm)	87.4 ± 11.1	85.4 ± 8.2	.277	1.94	−1.58	5.46
YBT composed score (cm)	92.2 ± 8.9	90.6 ± 8.0	.308	1.65	−1.54	4.84
AMEDA (AUC score)	.628 ± .042	.603 ± .040	**.003**	.024	**.009**	**.04**
**Muscle strength measurements**						
Knee extension (N)	251.2 ± 44.3	227.4 ± 52.7	**.017**	23.80	**4.32**	**43.28**
Knee flexion (N)	125.3 ± 18.5	116.2 ± 20.5	**.022**	9.06	**1.34**	**16.78**

*BMI* Body mass index, *YBT anterior* Y balance test in the anterior direction, *YBT Post. Med.* Y balance test in the posterio-medial direction, *YBT Post. Lat.* Y balance test in the postero-lateral direction, *AMEDA* Active Movement Extent Discrimination Apparatus

The proportion of dancers with no anomaly (74/132 dancers, 56.1%) was significantly higher compared with dancers with both GJH and scoliosis (34/132 dancers, 25.8%) (*p* < .001). Four dancers (3.0%) had only scoliosis and no GJH, and twenty dancers (15.2%) had only GJH with no scoliosis. Comparing the group of dancers with both GJH and scoliosis to the group of dancers with no GJH and no scoliosis, the first group had significantly reduced dynamic postural balance in the anterior direction (*p* = .023), significantly reduced proprioception ability (*p* < .001), significantly weaker knee extensors (*p* = .036), and significantly weaker knee flexors (*p* = .040) (Table 4). There were no statistically significant differences between those with and those without both anomalies on any of the other outcome measures.

**Table 4:** Anthropometric parameters, postural balance and proprioception muscle strength measurements in dancers with both anomalies and with no-anomalies

	Dancers with no anomalies^a^ (***n*** = 74)	Dancers with both anomalies^a^ (***n*** = 34)	***p*** value	Mean Difference	95% CI	
**Anthropometric parameters**					Lower	Upper
BMI (Kg/m^2^)	18.9 ± 2.5	19.8 ± 2.3	.107	−.83	−1.85	.18
**Postural balance and proprioception**						
YBT Anterior (cm)	57.9 ± 5.3	55.6 ± 5.6	**.046**	2.36	**.047**	**4.66**
YBT Post. Med. (cm)	87.4 ± 9.6	85.4 ± 8.5	.294	1.97	−1.74	5.69
YBT Post. Lat. (cm)	88.4 ± 10.8	85.4 ± 9.7	.165	2.95	−1.23	7.15
YBT composed score (cm)	91.8 ± 9.4	89.9 ± 7.9	.303	1.83	−1.67	5.34
AMEDA (AUC score)	.639 ± .037	.596 ± .036	**<.001**	.043	**.027**	**.058**
**Muscle strength measurements**						
Knee extension (N)	250.5 ± 46.1	227.5 ± 53.2	**.036**	22.97	**1.56**	**44.37**
Knee flexion (N)	125.7 ± 19.0	117.0 ± 20.1	**.040**	8.69	**.40**	**16.98**

^a^GJH and Scoliosis. *BMI* Body mass index, *YBT anterior* Y balance test in the anterior direction, *YBT Post. Med.* Y balance test in the posterio-medial direction, *YBT Post. Lat.* Y balance test in the postero-lateral direction, *AMEDA* Active Movement Extent Discrimination Apparatus

The total *n* = 108 presented in that table includes only dancers with both anomalies or with no-anomalies. Other 24 dancers had only one anomaly

Table 5 shows the frequency (95% CI) of dancers with unilateral PFP, bilateral PFP, and no PFP. Significant differences were found in the proportions of dancers with unilateral PFP, dancers with bilateral PFP, and dancers with no PFP (*p* < .001). Among the GJH dancers, 59.3% (32/54) suffered bilateral PFP and only 20.4% (11/54) had no PFP. Those percentages are significantly different from dancers with no GJH who suffered bilateral PFP [30.8% (24/78)] with no PFP [48.7% (38/78)] (*p* = .001). The frequency of PFP among scoliotic dancers (71.1% with bilateral PFP and 13.2% with no PFP) was found to be significantly different from the frequency of PFP in non-scoliotic dancers (30.9% with bilateral PFP and 46.8% with no PFP) (*p* < .001). Finally, in dancers with both GJH and scoliosis, 73.5% (25/34) suffered from bilateral PFP and only 8.8% (3/34) had no PFP. That frequency was significantly different compared with 29.7% (22/74) and 48.6% (36/74), respectively, in dancers with no anomalies (*p* < .001) (Table 5).

**Table 5:** Patellofemoral pain (PFP) frequencies in dancers with and with no-GJH, dancers with and with no-scoliosis and dancers with and with no-anomaly

		95% CI			95% CI	
	**Dancers with no- GJH (*n*** **= 78)**	Lower	Upper	**Dancers with GJH (*n*** **= 54)**	Lower	Upper
No-PFP	(38) 48.7%	37.8%	59.7%	(11) 20.4%	11.3%	32.5%
Unilateral PFP	(16) 20.5%	12.7%	30.4%	(11) 20.4%	11.3%	32.5%
Bilateral PFP	(24) 30.8%	21.4%	41.6%	(32) 59.3%	46.0%	71.6%
	**Dancers with no-scoliosis (*n*** **= 94)**			**Dancers with scoliosis (*n*** **= 38)**		
No-PFP	(44) 46.8%	36.9%	56.9%	(5) 13.2%	5.2%	26.5%
Unilateral PFP	(21) 22.3%	14.8%	31.5%	(6) 15.8%	6.9%	29.7%
Bilateral PFP	(29) 30.9%	22.2%	40.7%	(27) 71.1%	55.5%	83.5%
	**Dancers with no anomalies**^a^ **(*n*** **= 74)**^b^			**Dancers with both anomalies**^**a**^ **(*n*** **= 34)**^**b**^		
No-PFP	(36) 48.6%	37.5%	59.9%	(3) 8.8%	2.5%	21.7%
Unilateral PFP	(16) 21.6%	13.4%	32.0%	(6) 17.6%	7.7%	32.8%
Bilateral PFP	(22) 29.7%	20.2%	40.8%	(25) 73.5%	57.2%	86.0%

^a^GJH and Scoliosis

^b^Dancers with only one anomaly are not included

## Discussion

The main findings of the current study were that 25.8% of the dancers had both GJH and scoliosis. Dancers with both anomalies had significantly reduced dynamic postural balance, reduced proprioception ability, weaker knee extensors and flexors, and a high prevalence of bilateral and unilateral PFP, compared with dancers with no anomalies. GJH has been reported in the literature as a common phenomenon among the young female population as a whole, and among athletes and dancers in particular [31]. Reports on the prevalence of GJH in the general population show a range from 0.6 to 31.5% [39], with the prevalence reported for dancers exceeding 97% [40]. The prevalence of GJH that was found among our dancers (40.9%) is high compared with previous studies on adolescent dancers at a similar age range. Yet, the low percentage (24.2%) that was reported by Steinberg and colleagues [41] was found to increase with age up to 34.6% among dancers around the pubertal ages [41]. The prevalence of scoliosis in our study (28.8%) is similar to that reported in other literature assessing paediatric dancers (22–30%) [14, 16, 18]. It should be noted that scoliosis has already been noted in around one-fifth of the dancers at the age of 9, a prevalence that increased slightly to one quarter of the dancers at the age of 16 years old [14]. Probably, the high prevalence of dancers with GJH and dancers with scoliosis in the present study should be attributed to selection of body type by the dance teachers and self-selection by the dancers themselves [6, 7, 18].

The presense of adolescent girls with both scoliosis and GJH has barely been explained in the literature. In non-dancer children and adolescents, Czaprowski and colleagues [42] found GJH in 51% of the children (aged 9–18 years) diagnosed with scoliosis, compared with 19% in those without scoliosis [42]. The authors could not conclude whether these results represent a statistical phenomenon or whether a cause-result relation could be drawn, and suggested further studies to explain these results. In addition, GJH was noted in non-dancer adolescents that also had disturbances of posture, particularly scoliotic posture and sway-back posture [24, 43]. In young dancers, anomalies such as genu-recurvatum, splay foot, and hallux valgus were found more frequently among scoliotic dancers than non-scoliotic dancers [14]; and, GJH, flexibility and body composition were reported to be more common in young dancers with scoliosis [16, 18, 44]. GJH was suggested as a contributing factor in scoliosis [45]. The GJH dancers mostly had altered collagen structure, which may compromise spinal integrity and predispose the dancer to spinal instability and the development of scoliosis [45].

Moreover, in the current study, almost all dancers with both GJH and scoliosis suffered from PFP. A literature review showed that in the general population, PFP is more common in adolescent individuals with GJH compared to adolescents with no GJH [24]. Particularly in dancers, a higher prevalence of musculoskeletal conditions was found in dancers with GJH compared to dancers with no GJH [1]. Adolescent dancers (12–14 years old) with PFP had a greater prevalence of scoliosis compared with dancers with no-PFP [26]. An explanation for the high prevalence of PFP in dancers with GJH and with scoliosis might be attributed to the delayed maturation of the adolescent dancer with those anomalies [16, 44]. It is known that a high prevalence of adolescent dancers manifested a late age of menarche, lower body weight, and disturbance of the regular ovulatory cycles with a delayed growth-spurt period – parameters that might effect and might advance the occurrence of musculoskeletal conditions during growth and the maturation period [1, 16, 46–49]. In the general non-dancer adolescents, a known relationship exists between growth in adolescence and development of a spinal deformity. It was shown that a rapid increase in spinal height at the time of the pubertal growth spurt causes an increase in spinal curvature [50]. Excessive mechanical loading may affect bone growth plates (mainly the metaphysis), and hence be connected to scoliosis progression [51]. Most young dancers with scoliosis and with GJH tend to have late maturation [14, 16, 21]; thus, all the repetitive, highly-demanding activity of the immature hyperextended and curvature skeleton might affect the bone growth plates and contribute to the progression of conditions such as knee pain [14, 16, 21, 52, 53]. Furthermore, malalignment of the bones and joints of the lower limbs and the spine of dancers with scoliosis and GJH might distort the direction of forces passing across the joints, and place them at higher risk to suffer PFP [54–56]. Any lower limb or spinal deformities may change the normal biomechanics and alter the forces to the knee [57], and that in turn may increase the risk for knee conditions [54, 58, 59].

Dancers with both phenomena had significantly reduced strength ability in the muscles around the knee. Yusof [57] suggested that reduced knee muscle strength in scoliotic subjects may exaggerate the interference of the biomechanics of the knee, as well as restrict the normal movement of the spinal segments and the loads across the knee joints, with increased risk for PFP. Yet, no previous study measured knee strength in scoliotic young dancers. Considering GJH, similar to our results, Scheper et al. [9] showed that dancers with GJH manifested lower muscle strength. This reduced muscle strength can be explained by the difference in connective tissue and the increased amount of fragile elastic fibers, which resulted in inefficient force transfer through muscle fibers [60]. As the intense demands of the various dance positions require sufficient muscle strength [61–63], the hyper-lax joints require even more intensive muscle effort to maintain stability and to decrease the risk for musculoskeletal conditions [8, 9].

Proprioception ability and postural balance were found to be reduced in dancers with both phenomena. Sporadic research reports can be found on decreased proprioception and balance problems in subjects with scoliosis [64, 65]. It was explained that following immature central integration of the proprioceptive input of the scoliotic subjects, poorly adapted postural control may occur. In addition, scoliosis was previously found in adolescents with prolonged growth spurts [14]. During that growth period, coordination, proprioception, and posture parameters might decline, and the probability of developing injuries might increase [66]. It was proposed that pubertal dancers should improve their DPB and proprioception abilities in order to prevent injuries [36]. Reduced ankle proprioception in the frontal plane (inversion) was found in adolescents with spinal deformity [67, 68]. It was suggested that scoliosis may be related to both peripheral and central sensory system impairments. As the central sensory-reweighting mechanisms are less effective and the transformation of the sensory-orientation cues to the sensory organs are improper in scoliotic subjects, balance control instructions and proprioception ability might be inappropriate among that population [68]. Reduced joint proprioception was found in individuals with GJH [60], yet that was not measured in adolescent dancers. It was suggested that poor proprioception, especially during physical activities, is related to reduced muscle strength in GJH subjects, as muscle atrophy was found to result in a reduction of proprioceptive sensor density. Decreased ankle proprioception ability was previously found in young female dancers with PFP [27]. It was explained that any restricted/exaggerated ankle movements may affect the movements of the lower limb and may injure the patellofemoral complex in those dancers.

### Clinical implications

Dance teachers, dance experts, and physicians should develop screening programs to effectively identify dancers with scoliosis and GJH. In order to reduce the high risk of PFP among these dancers, it is important to develop specific exercises to cope with the demands of the anomalous dancers. Proprioceptive training and postural balance exercises may improve the correct alignment and the accurate range desired for each joint. Improved muscle strength, achieved by specific strength training, is required for improving the stabilization of dancers with GJH and for dancers with scoliosis – that is, for countering hyper-extension of the joints, for bettering aesthetic dance performance and for decreasing the risk of musculoskeletal conditions. Combined treatment based on muscle strengthening, increased proprioception acuity, and symmetric and balanced exercises should be considered when physiotherapy is planned. Future studies should seek answers to such questions as: “Do these two anomalies cause more injuries?”, and “Are these kinds of anomalies associated with success?”.

### Limitations of the study

The main limitation of the present study is its cross-sectional nature, with no control group. In addition, we could not control for parameters such as load/type of exercises, as the dancers were sampled from three schools and studied with different teachers. Other limitations include the fact that we measured only a very narrow age range of dancers (12–14 years); only female dancers participated in our study; and, only dancers who participated in a specific dance program were recruited to the study. Measurements of knee proprioception and for spinal proprioception are better than ankle proprioception assessments for dancers with PFP and for dancers with scoliosis, respectively.

## Conclusions

Girls who have GJH and scoliosis tend to choose dancing as their preferred sport, as most likely their bodies are better suited for this activity. The high prevalence of scoliosis in young dancers might be attributed to the relatively low body mass, delayed maturation, and the selection process. Dancers with both GJH and scoliosis had decreased knee muscle strength, reduced postural balance, and reduced proprioception ability, with a higher risk to experience patellofemoral pain. The main clinical implications of the current study are the need to reduce the risk of patellofemoral pain among these dancers by developing appropriate strength and stabilizing exercises combined with proprioceptive and postural balance training, with the goal of improving the correct alignment of the hyperextended and hypermobile joints and improving their supporting muscle strength and flexibility.

## Data Availability

The datasets used and/or analyzed during the current study are available from the corresponding author on reasonable request.

## Abbreviations

AMEDA: Active Movement Extent Discrimination Apparatus
AUC: Area Under the Curve
BMI: Body Mass Index
DPB: Dynamic Postural Balance
GJH: Generalized Joint Hypermobility
HHD: Hand-Held Dynamometry
ICC: Intra-Class Correlations
PFP: Patellofemoral Pain
VAS: Visual Analogue Scale
YBT: Y Balance Test

## References

[1] Briggs J, McCormack M, Hakim AJ, Grahame R (2009). Injury and joint hypermobility syndrome in ballet dancers--a 5-year follow-up. Rheumatology..

[2] Sundemo D, Hamrin Senorski E, Karlsson L, Horvath A, Juul-Kristensen B, Karlsson J (2019). Generalised joint hypermobility increases ACL injury risk and is associated with inferior outcome after ACL reconstruction: a systematic review. BMJ Open Sport Exerc Med.

[3] Grahame R (1990). The hypermobility syndrome. Ann Rheum Dis.

[4] Castori M, Tinkle B, Levy H, Grahame R, Malfait F, Hakim A (2017). A framework for the classification of joint hypermobility and related conditions. Am J Med Genet C: Semin Med Genet.

[5] Remvig L, Jensen DV, Ward RC (2007). Epidemiology of general joint hypermobility and basis for the proposed criteria for benign joint hypermobility syndrome: review of the literature. J Rheumatol.

[6] Hamilton WG, Hamilton LH, Marshall P, Molnar M (1992). A profile of the musculoskeletal characteristics of elite professional ballet dancers. Am J Sports Med.

[7] McCormack M, Briggs J, Hakim A, Grahame R (2004). Joint laxity and the benign joint hypermobility syndrome in student and professional ballet dancers. J Rheumatol.

[8] Foley EC, Bird HA (2013). Hypermobility in dance: asset, not liability. Clin Rheumatol.

[9] Scheper MC, de Vries JE, Juul-Kristensen B, Nollet F, Engelbert RH (2014). The functional consequences of generalized joint hypermobility: a cross-sectional study. BMC Musculoskelet Disord.

[10] Tinglea A, Bennetta O, Wallisa A, Palmera S (2018). The links between generalized joint laxity and the incidence, prevalence and severity of limb injuries related to physical exercise: a systematic literature review. Phys Ther Rev.

[11] Pacey V, Nicholson LL, Adams RD, Munn J, Munns CF (2010). Generalized joint hypermobility and risk of lower limb joint injury during sport: a systematic review with meta-analysis. Am J Sports Med.

[12] Weinstein SL, Dolan LA, Cheng JC, Danielsson A, Morcuende JA (2008). Adolescent idiopathic scoliosis. Lancet..

[13] Altaf F, Gibson A, Dannawi Z, Noordeen H (2013). Adolescent idiopathic scoliosis. Bmj..

[14] Steinberg N, Hershkovitz I, Peleg S, Dar G, Masharawi Y, Zeev A (2013). Morphological characteristics of the young scoliotic dancer. Phys Ther Sport.

[15] Omey ML, Micheli LJ, Gerbino PG (2000). 2nd. Idiopathic scoliosis and spondylolysis in the female athlete. Tips for treatment. Clin Orthop Relat Res.

[16] Warren MP, Brooks-Gunn J, Hamilton LH, Warren LF, Hamilton WG (1986). Scoliosis and fractures in young ballet dancers. Relation to delayed menarche and secondary amenorrhea. N Engl J Med.

[17] Warren M, Gunn B, Hamilton L, Warren LF, Hamilton W (2007). Dance anatomy and kinesiology: principles and exercises for improving technique and avoiding common injuries: Champaign; human kinetics.

[18] Longworth B, Fary R, Hopper D (2014). Prevalence and predictors of adolescent idiopathic scoliosis in adolescent ballet dancers. Arch Phys Med Rehabil.

[19] Ko KJ, Kang SJ (2017). Effects of 12-week core stabilization exercise on the cobb angle and lumbar muscle strength of adolescents with idiopathic scoliosis. J Exerc Rehabil.

[20] Dufvenberg M, Adeyemi F, Rajendran I, Oberg B, Abbott A (2018). Does postural stability differ between adolescents with idiopathic scoliosis and typically developed? A systematic literature review and meta-analysis. Scoliosis Spinal Disord.

[21] Gamboa J, Roberts L, Maring J, Fergus A (2008). Injury patterns in elite Preprofessional ballet dancers and the utility of screening programs to identify risk characteristics. J Orthop Sports Phys Ther.

[22] Kenanidis E, Potoupnis ME, Papavasiliou KA, Sayegh FE, Kapetanos GA (2008). Adolescent idiopathic scoliosis and exercising: is there truly a liaison?. Spine (Phila Pa 1976).

[23] Kenanidis EI, Potoupnis ME, Papavasiliou KA, Sayegh FE, Kapetanos GA (2010). Adolescent idiopathic scoliosis in athletes: is there a connection?. Phys Sportsmed.

[24] Murray KJ (2006). Hypermobility disorders in children and adolescents. Best Pract Res Clin Rheumatol.

[25] Steinberg N, Stern M, Tenenbaum S, Blankstein A, Zeev A, Siev-Ner I (2018). Ultrasonography and clinical examination of knee injuries in pre- and post- menarche dancers. Res Sports Med.

[26] Steinberg N, Tenenbaum S, Hershkovitz I, Zeev A, Siev-Ner I (2017). Lower extremity and spine characteristics in young dancers with and without patellofemoral pain. Res Sports Med.

[27] Steinberg N, Tenenbaum S, Waddington G, Adams R, Zakin G, Zeev A (2020). Unilateral and bilateral patellofemoral pain in young female dancers: associated factors. J Sports Sci.

[28] Siev-Ner I, Stern MD, Tenenbaum S, Blankstein A, Zeev A, Steinberg N (2018). Ultrasonography findings and physical examination outcomes in dancers with and without patellofemoral pain. Phys Sportsmed.

[29] Tanner JM (1981). Growth and maturation during adolescence. Nutr Rev.

[30] Steinberg N, Hershkovitz I, Zeev A, Rothschild B, Siev-Ner I (2016). Joint hyper-mobility and joint range of motion in young dancers. J Clin Rheumatol.

[31] Gannon LM, Bird HA (1999). The quantification of joint laxity in dancers and gymnasts. J Sports Sci.

[32] Adams W (1865). Lectures on the pathology and treatment of lateral and other forms of curvature of the spine. Br Foreign Med Chir Rev.

[33] Magee DJ (2013). Orthopedic physical assessment, 6th edition: Elsevier health sciences.

[34] Steinberg N, Tenenbaum S, Stern M, Zeev A, Siev-Ner I (2019). Patellofemoral pain, body morphology and alignment in female pubertal dancers: one-year follow-up. J Sports Sci.

[35] Coughlan GF, Fullam K, Delahunt E, Gissane C, Caulfield BM (2012). A comparison between performance on selected directions of the star excursion balance test and the Y balance test. J Athl Train.

[36] Steinberg N, Adams R, Tirosh O, Karin J, Waddington G (2019). Effects of textured balance board training in adolescent ballet dancers with ankle pathology. J Sport Rehabil.

[37] Waddington G, Adams R (2003). Football boot insoles and sensitivity to extent of ankle inversion movement. Br J Sports Med.

[38] Steinberg N, Tenenbaum S, Waddington G, Adams R, Zakin G, Zeev A (2020). Isometric exercises and somatosensory training as intervention programmes for patellofemoral pain in young dancers. Eur J Sport Sci.

[39] Seckin U, Tur BS, Yilmaz O, Yagci I, Bodur H, Arasil T (2005). The prevalence of joint hypermobility among high school students. Rheumatol Int.

[40] Day H, Koutedakis Y, Wyon MA (2011). Hypermobility and dance: a review. Int J Sports Med.

[41] Steinberg N, Hershkovitz I, Zeev A, Rothschild B, Siev-Ner I (2016). Joint hypermobility and joint range of motion in young dancers. J Clin Rheumatol.

[42] Czaprowski D, Kotwicki T, Pawlowska P, Stolinski L (2011). Joint hypermobility in children with idiopathic scoliosis: SOSORT award 2011 winner. Scoliosis..

[43] Adib N, Davies K, Grahame R, Woo P, Murray KJ (2005). Joint hypermobility syndrome in childhood. A not so benign multisystem disorder?. Rheumatology..

[44] Hamilton D, Aronsen P, Loken JH, Berg IM, Skotheim R, Hopper D (2006). Dance training intensity at 11–14 years is associated with femoral torsion in classical ballet dancers. Br J Sports Med.

[45] Binns M (1988). Joint laxity in idiopathic adolescent scoliosis. J Bone Joint Surg (Br).

[46] Myer GD, Ford KR, Paterno MV, Nick TG, Hewett TE (2008). The effects of generalized joint laxity on risk of anterior cruciate ligament injury in young female athletes. Am J Sports Med.

[47] Rietveld AB (2013). Dancers’ and musicians’ injuries. Clin Rheumatol.

[48] Roussel N, De Kooning M, Schutt A, Mottram S, Truijen S, Nijs J (2013). Motor control and low back pain in dancers. Int J Sports Med.

[49] Sanches SB, Oliveira GM, Osorio FL, Crippa JA, Martin-Santos R (2015). Hypermobility and joint hypermobility syndrome in Brazilian students and teachers of ballet dance. Rheumatol Int.

[50] Busscher I, Wapstra FH, Veldhuizen AG (2010). Predicting growth and curve progression in the individual patient with adolescent idiopathic scoliosis: design of a prospective longitudinal cohort study. BMC Musculoskelet Disord.

[51] Busscher I, Kingma I, Wapstra FH, Bulstra SK, Verkerke GJ, Veldhuizen AG (2011). The value of shoe size for prediction of the timing of the pubertal growth spurt. Scoliosis..

[52] Steinberg N, Siev-Ner I, Peleg S, Dar G, Masharawi Y, Hershkovitz I (2008). Growth and development of female dancers aged 8-16 years. Am J Hum Biol.

[53] Steinberg N, Stern M, Tenenbaum S, Blankstein A, Siev-Ner I (2017). Ultrasonography and clinical examination for knee injuries and morphology of pre- and post-menarche dancers. Res Sports Med.

[54] Hurwitz DE, Ryals AB, Case JP, Block JA, Andriacchi TP (2002). The knee adduction moment during gait in subjects with knee osteoarthritis is more closely correlated with static alignment than radiographic disease severity, toe out angle and pain. J Orthop Res.

[55] Hewett TE, Myer GD, Ford KR (2004). Decrease in neuromuscular control about the knee with maturation in female athletes. J Bone Joint Surg Am.

[56] Schmitz RJ, Shultz SJ, Nguyen AD (2009). Dynamic valgus alignment and functional strength in males and females during maturation. J Athl Train.

[57] Yusof MI, Shaharudin S, Sivalingarajah P (2018). Does vertical ground reaction force of the hip, knee, and ankle joints change in patients with adolescent idiopathic scoliosis after spinal fusion?. Asian Spine J.

[58] Ahonen J (2008). Biomechanics of the foot in dance: a literature review. J Dance Med Sci.

[59] Hunter DJ, Niu J, Felson DT, Harvey WF, Gross KD, McCree P (2007). Knee alignment does not predict incident osteoarthritis: the Framingham osteoarthritis study. Arthritis Rheum.

[60] Scheper M, Rombaut L, de Vries J, De Wandele I, van der Esch M, Visser B (2017). The association between muscle strength and activity limitations in patients with the hypermobility type of Ehlers-Danlos syndrome: the impact of proprioception. Disabil Rehabil.

[61] Liederbach M, Dilgen FE, Rose DJ (2008). Incidence of anterior cruciate ligament injuries among elite ballet and modern dancers: a 5-year prospective study. Am J Sports Med.

[62] Myer GD, Ford KR, McLean SG, Hewett TE (2006). The effects of plyometric versus dynamic stabilization and balance training on lower extremity biomechanics. Am J Sports Med.

[63] Orishimo KF, Kremenic IJ, Pappas E, Hagins M, Liederbach M (2009). Comparison of landing biomechanics between male and female professional dancers. Am J Sports Med.

[64] Byl NN, Holland S, Jurek A, Hu SS (1997). Postural imbalance and vibratory sensitivity in patients with idiopathic scoliosis: implications for treatment. J Orthop Sports Phys Ther.

[65] Le Berre M, Guyot MA, Agnani O, Bourdeauducq I, Versyp MC, Donze C (2017). Clinical balance tests, proprioceptive system and adolescent idiopathic scoliosis. Eur Spine J.

[66] Richardson M, Liederbach M, Sandow E (2010). Functional criteria for assessing pointe-readiness. J Dance Med Sci.

[67] Fortin C, Pialasse JP, Knoth IS, Lippe S, Duclos C, Simoneau M (2019). Cortical dynamics of sensorimotor information processing associated with balance control in adolescents with and without idiopathic scoliosis. Clin Neurophysiol.

[68] Simoneau M, Mercier P, Blouin J, Allard P, Teasdale N (2006). Altered sensory-weighting mechanisms is observed in adolescents with idiopathic scoliosis. BMC Neurosci.

